# Global mechanisms to improve affordability of high-priced medicines: the role of the WHO model lists of essential medicines

**DOI:** 10.1080/20523211.2025.2601935

**Published:** 2026-01-09

**Authors:** Kristina Jenei

**Affiliations:** Department of Health Policy, London School of Economics and Political Science, London, UK

**Keywords:** Essential medicines, access to medicines, world health organization, pharmaceutical pricing, economics

## Abstract

**Introduction:**

The scope of the WHO Model Lists of Essential Medicines (EML) has evolved from a tool for resource-constrained countries to a global benchmark that includes several high-priced, patented medicines. The addition of high-priced medicines has sparked a recent debate about whether affordability should be more explicitly considered in WHO EML listing decisions.

**Discussion and Analysis:**

Currently, WHO must face the difficult task of balancing clinical benefits with the economic realities of the current pharmaceutical market. As such, a key question arises: Should the WHO EML consider prices at the time of listing, or does EML inclusion serve as a catalyst for price reductions through targeted mechanisms post-listing? This analysis explores the complexities of including high-priced medicines on the Model Lists.

**Analysis:**

Challenges include market and regulatory exclusivities, marginal clinical benefits, difficulties with applying cost-effectiveness analyses globally, and the disconnect between production costs and market prices. Several mechanisms that could facilitate post-listing price reductions are reviewed, including voluntary and compulsory licenses, pooled procurement, WHO prequalification, price transparency, and political advocacy. These mechanisms are frequently referenced in EML recommendations and the academic literature but have not been examined together.

**Conclusion:**

This analysis provides insights to inform ongoing WHO reforms and a foundation for future research evaluating the downstream economic impacts of the WHO EML on access to medicines worldwide.

## Introduction

Since 1977, the World Health Organization (WHO) has reinforced the concept of essential medicines through the biannual publication of the Model Lists of Essential Medicines (EML or Model Lists), serving as an important tool to guide countries select high-benefit, cost-effective medicines to address the priority needs of their populations (Wirtz et al., 2017). Once a tool for resource-constrained countries, the scope of the Model Lists has evolved to include several high-priced medicines (Brhlikova et al., 2023). A recent debate has emerged about whether price and affordability should be more explicitly considered in listing decisions, especially given the challenges of ensuring equitable access across diverse healthcare systems (Jenei et al., 2022; Piggott, Moja, Huttner, et al., 2024; Wirtz et al., 2024). Recognising these issues, WHO recently established a Technical Advisory Group on Pricing Policies for Medicines (World Health Organization, 2023) and announced a reform process to revise the selection procedures for the Model Lists (i.e. revising criteria, and evaluation processes) (World Health Organization, 2024).

**Figure 1.**
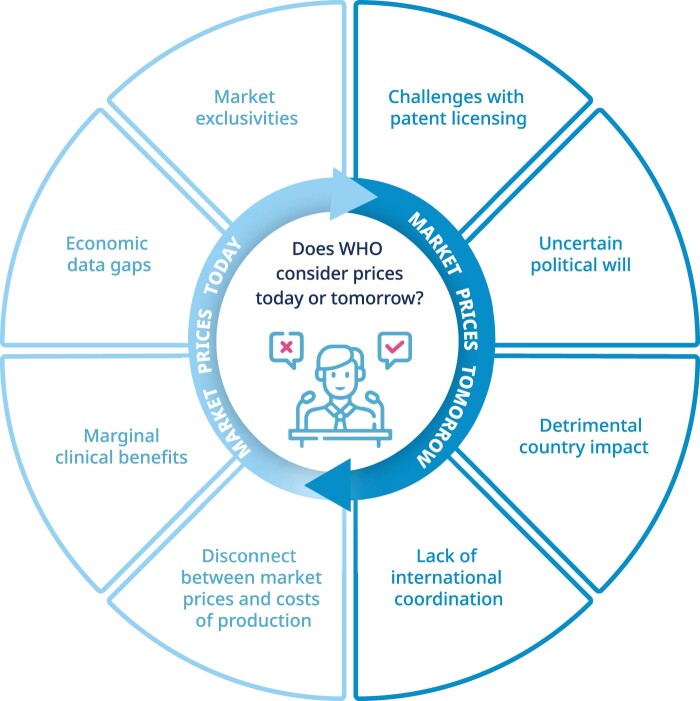
Uncertainties in WHO EML decision-making for high-priced medicines. Figure 1 presents an overview of the uncertainties facing WHO EML decisions on high-priced medicines. On the left, key challenges such as market exclusivities, economic data gaps, and marginal clinical benefits are highlighted. On the right, potential mechanisms for reducing prices, such as voluntary licensing, pooled procurement, and political advocacy, are mapped. Together, these illustrate the tension between current barriers and the uncertain effectiveness of mechanisms aimed at improving future affordability and access. Commissioned by the author and created by a professional medical illustrator (contact details in acknowledgement); the author retains full rights to use and publish this figure.

**Figure 2.**
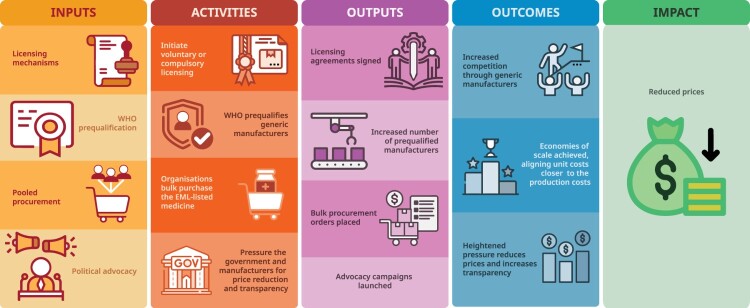
Hypothetical framework of post-listing mechanisms to reduce medicine prices. Figure 2 illustrates a hypothetical framework of post-listing mechanisms that may reduce prices of high-cost medicines included on the WHO EML. The figure outlines how interventions, such as licensing, prequalification, pooled procurement, and political advocacy, may lead to specific activities, outputs, and outcomes that support price reductions by increasing competition, achieving economies of scale, and applying pressure for price transparency. Commissioned by the author and created by a professional medical illustrator (contact details in acknowledgement); the author retains full rights to use and publish this figure.

A key question arises: Should the WHO EML consider prices at the time of listing, or does EML inclusion serve as a catalyst for downstream price reductions through targeted post-listing mechanisms? These pre- and post-listing uncertainties have created substantial difficulties in recommendations for essential medicines, especially for high-priced therapeutics with unclear downstream affordability (Figure 1). Originally, costs were a major criterion in listing decisions as affordability was central to the objectives of the Model Lists (Laing et al., 2003). However, in 2001, WHO revised the criteria and removed the ‘total costs’ and ‘patent status’, thus prioritising clinical benefit in the selection of essential medicines (cost-effectiveness is considered within therapeutic alternatives) (World Health Organization, 2001) Affordability became a desired outcome rather than a precondition for inclusion (Jenei, 2025). This approach has been most prominently associated with the 2001 addition of several widely patented antiretrovirals and the 2015 inclusion of medicines for cancer, diabetes, tuberculosis, and hepatitis C (Figure 2) (Magrini et al., 2015).

Resolving this debate is closely tied to the effectiveness of downstream mechanisms that may reduce prices. While the inclusion of a medicine on the Model Lists may support broader access efforts, to my knowledge, there is currently no evidence that establishes a causal link between EML listing and subsequent price reductions (Wirtz et al., 2017). Nevertheless, several post-listing mechanisms have been theorised to facilitate market competition and reduce prices after EML listing (Figure 2). The relationship between WHO EML inclusion and reduced prices is complex, as highlighted in Expert Committee recommendations (Barber, 2023) and the wider academic literature (Supplemental Material). However, the connection is unclear, and the dialogue is fragmented.

The purpose of this Analysis article was to review the existing literature to identify challenges associated with listing high-priced medicines on the WHO EML and map global-level mechanisms that may support post-listing price reductions. While several national strategies exist, such as managed entry agreements, procurement strategies, national essential medicines lists, this article focuses on global governance mechanisms, given the emphasised role of the WHO EML in countries where these national institutions may be absent or underdeveloped. In the unlikely scenario that prices spontaneously decrease, this analysis offers insights for ongoing WHO procedural reforms for selecting medicines and a foundation for future research to examine its impact.

This analysis is informed by a structured narrative review of peer-reviewed and grey literature, including WHO publications, IRIS, and academic databases. Search terms included combinations of ‘Essential Medicines’, ‘Essential Drugs’, ‘World Health Organization’, ‘WHO’, and ‘price’ or ‘cost’. Initial searches were conducted in IRIS and on the WHO EML website, including queries of the Technical Report Series for terms such as ‘price’, ‘cost’, or ‘expenses’. These documents were used to understand how WHO describes pricing challenges and to ensure alignment with ongoing reforms The challenges discussed in this paper were identified through a thematic analysis of WHO documents, which highlighted a set of recurring issues relating to affordability and access.

For peer-reviewed literature, a structured search was conducted using OVID Embase, OVID Medline, CINAHL (nursing and allied health), PsycINFO Ovid (psychology and behavioral science, for mental health), Global Health Ovid (targeted search for developing countries). This yielded 1,577 results, which were filtered to 202 papers for full-text review based on relevance to the WHO EML and high-priced medicines. Inclusion criteria prioritised literature discussing global WHO processes, challenges of high-priced medicines, and pricing mechanisms. Due to space constraints, not all references could be cited in the manuscript. However, a list of these references, organised by topic, is available in Supplemental Table S1. This list could also serve as an additional resource for future research.

## Discussion

### Challenges in ensuring affordability

#### Market exclusivities and patent protections

Patent protections are associated with high prices and can be significant barriers to affordability given market exclusivity prevents generic competition. In 1995, the World Trade Organization Agreement on Trade-Related Aspects of Intellectual Property Rights (TRIPS) established minimum standards for intellectual property rights, including pharmaceutical patents (Figure 2). The 2005 transition period marked a turning point for many countries (although least developed countries benefited from extended grace periods that have since been renewed). Global generic supply nonetheless shifted substantially after 2005 given TRIPS compliance in countries such as India, which had been a major source of affordable generics.

In countries with strong patent enforcement, manufacturers can launch drugs at higher prices. Between 2008 and 2021, launch prices increased 20% per year in the United States, with some medicines now priced above US$150,000 annually (Rome et al., 2022). In some markets with limited competition, such as oncology, prices continue to rise by 10% per year despite within-class competition (Howard et al., 2015). Market exclusivity delays the entry of more affordable generics which could reduce prices by 20-80% (Nguyen et al., 2022). In addition to patents, regulatory protections, such as data exclusivities prolong market dominance by the originator. Without competition, prices remain high, limiting the potential impact of EML listing and creating uncertainties about the post-listing pathway to affordability.

#### Disconnect between market prices and production costs

Pharmaceuticals are often priced higher than the costs required to produce them (Hill et al., 2018). For example, in 2015, six oral direct-acting antivirals (DAAs) were added to the Model Lists for hepatitis C despite high prices given their public health importance. At the time, these medicines were prohibitively expensive(the median price of sofosbuvir in the US was US$40,502 (Barber et al., 2020).) However, the clinical benefit was considerable (e.g. sustained virological responses greater than 90% with fewer adverse events compared to interferon-based regimens) (World Health Organization, 2015). In the recommendation, the Expert Committee noted that costs of production were substantially lower at US$28 (including tax and a 10% profit margin) (Barber et al., 2020).

Studies using the same costing algorithm have estimated other high-priced cancer medicines on the WHO EML could also be produced affordably, such as lenalidomide ($2.55 per month), afatinib ($8.85 per month), and abiraterone ($60.97 per month) (T’Hoen et al., 2019). This is especially relevant in LMICs, where prices can be higher due to limited purchasing power, and the absence of government mechanisms to lower prices (e.g. health technology assessment, managed entry agreements, procurement strategies) (Cameron et al., 2009). Even generic chemotherapies can incur catastrophic expenditure in resource-constrained settings (Fundytus et al., 2021).

Pharmaceutical companies argue that high prices are necessary to recoup research and development (R&D) investments (including the costs of failed trials) (International Federation of Pharmaceutical Manufactorers & Associations, 2019). One study found that an optimal share allocated towards manufacturers to incentivise innovation, while minimising burden to health systems, should be approximately 22% (Woods et al., 2024). Yet, when evaluating cancer drugs reimbursed by the UK healthcare system, the actual share directed towards manufacturers was as high as 260% (Woods et al., 2024). The tension between high prices versus low production costs, large versus modest clinical benefits, and rising public health burdens versus rare disease priorities is central to WHO EML deliberations. In the case of DAAs, their inclusion may have catalysed price reduction mechanisms (e.g. voluntary licenses) that expanded treatment access and supported global hepatitis C elimination efforts.

#### Economic data gaps and marginal clinical benefits

High prices are complicated by the disconnect between costs and incremental health benefits. The ethos of the WHO EML is to recommend high-benefit medicines, irrespective of costs (cost-effectiveness is considered between therapeutic alternatives). High-priced medicines may still demonstrate favourable cost-effectiveness when clinical benefits are large enough to justify expenses. For example, imatinib was added in 2015 for chronic myeloid leukaemia approaching the end of market exclusivity in several jurisdictions. At the time, the price of imatinib was US$10,000 given the absence of generic competition (Cole & Dusetzina, 2018). Clinical benefit was substantial, improving survival rates from 6% to 87% (Kantarjian et al., 2012). Given these benefits, the incremental cost-effectiveness remained within conventional willingness-to-pay thresholds(World Health Organization, 2015).

However, medicines like imatinib are exceptions. Most cancer medicines offer modest gains, with median survival benefits estimated at solely two to four months (Schnog et al., 2021). Simultaneously, the burden of cancer is rising. Low and middle-income countries (LMICs) account for 57% of new cancer cases and 65% of deaths globally (Bamodu & Chung, 2024). In response, WHO established a Cancer Medicines Working Group (CMWG) and introduced extra criteria for cancer medicines inclusion on the WHO EML (Jenei et al., 2022). However, implementation has been challenging. For example, in October 2022, Novartis signed a voluntary licence with the Medicines Patent Pool (MPP) for nilotinib (a second-line treatment for CML resistant to imatinib). Yet, given patent expiry was imminent (July 2023), and the licence covered limited territories, the MPP advisory group was ‘unclear what the success factors were given the limited territory and low demand’ (Medicines Patent Pool, 2022). Access issues extend beyond oncology. Several insulin analogues have been added to the WHO EML since 2021 despite persistent concerns about high prices and the concentrated market power of the three dominant manufacturers (Beran et al., 2025). The recent inclusion of GLP-1 agonists raises similar concerns for countries with limited fiscal space (World Health Organization, 2025). These cases highlight that inclusion alone does not resolve structural price barriers, and they reinforce the need for meaningful transparency in global pricing.

While WHO reviews cost-effectiveness analyses (CEA) in applications, there are several issues when applying these estimates at a global level. First, CEAs are context specific. These analyses are often conducted in high-income countries with stable infrastructure, budgets, and purchasing power. In LMICs, healthcare systems, drug prices, and delivery infrastructure vary considerably, limiting the relevance of CEA findings. Second, drug prices vary across countries and are often not transparent. An analysis of the prices of dolutegravir for HIV treatment found wide variation in price across 52 countries unrelated to burden of disease, and gross domestic product per capita, nor by grouping countries by similar income levels (Sim & Hill, 2018). Third, the utility of CEA is further complicated by WHO’s unique role: it is not a payer and does not make reimbursement decisions but instead provides normative guidance for a diverse range of countries. As such, WHO must weigh broader criteria beyond traditional cost-effectiveness thresholds, such as feasibility, affordability, and public health impact.

### Global mechanisms that may improve affordability

#### Licensing strategies to address patent-related price barriers

One of the most direct ways to address affordability issues caused by patent protections is with voluntary or compulsory licenses (Figure 2). Both mechanisms are enabled by the TRIPS Agreement flexibilities, which allow either governments (in the case of compulsory licenses) or patent holders (in the case of voluntary licenses) to authorise generic production of patented medicines. While compulsory licenses typically require a justification grounded in public health interest, voluntary licenses may be issued by patent holders for commercial or strategic reasons.

Voluntary licenses are agreements where the patent holder grants permission to other manufacturers to produce and sell a patented medicine, typically for lower prices in specific countries. These agreements may be arranged bilaterally or via mechanisms such as the MPP but depend on the willingness of the patent holder to share rights. Bilateral voluntary licences may be exclusive or limited to a small number of licensees, whereas licences negotiated through the MPP are non-exclusive by design, allowing multiple manufacturers to participate. Importantly, voluntary licences had already been issued before the establishment of the MPP. For example, South Africa scaled up antiretroviral therapy in the public sector in 2004 using generic products licensed by GSK and Boehringer-Ingelheim, alongside non-enforcement agreements such as Bristol-Myers Squibb’s pledge on stavudine. While voluntary licences aim to expand access, they often include geographic or supply constraints that limit their reach. Some exclude upper-middle-income countries with high disease burdens or restrict the number of licensees, therefore reducing competition and access. Companies implement tiered or differential pricing strategies, but these are inconsistently applied, and their effectiveness is limited by external reference pricing and challenges in maintaining market boundaries, although the extend of this effect is contested (Danzon, 2018).

While the MPP can negotiate licenses on medicines excluded from the EML, the organisation has aligned its activities based on the WHO EML and guidelines since its inception in 2010 (Medicines Patent Pool, 2023). Explaining the timing between MPP licences and EML listing is complex. For example, the MPP negotiated a voluntary licence for dolutegravir (DTG) in 2014, very early and in parallel with evolving WHO guideline recommendations. One challenge is that EML applications may be submitted by a range of actors, and the Expert Committee only meets every two years. In addition, voluntary licensing and subsequent generic entry require time. Against this backdrop, the inclusion of DTG on the 2017 EML (based on a 2016 application) was not unusual. Instead of immediately listing several high-priced medicines, the Expert Committee has at times flagged potential candidates for MPP licensing prior to EML inclusion (e.g. osimertinib and enzalutamide in 2021) (World Health Organization, 2021). This hesitation may be explained as biologics are more complex to manufacture and have not led to the same price reductions of generic medicines (although there are exceptions in some markets with strong competition, such as India) (Morin et al., 2023). The MPP has recently expanded its mandate to biotherapeutics, including select cancer medicines (Morin et al., 2023). However, the feasibility of large-scale expansion remains uncertain given technical barriers and limited patent holder participation.

Compulsory licensing allows governments to override patent protections without the consent of the patent holder in the interest of public health (World Trade Organization, 2024). While there is no formal link between the WHO EML and TRIPS flexibilities, listing a medicine as essential can strengthen the legal and moral rationale for issues a compulsory license (Jenei, 2025). For example, a 1999 WTO Ministerial working paper proposed allowing compulsory licenses for medicines on the Model Lists. In another example, after imatinib was added to the WHO EML, the Colombian Minister of Health contacted WHO for guidance on pursuing a compulsory license for the medicine (Kieny, 2016). Alternatively, pharmaceutical companies have cited the absence of EML inclusion to argue against licensing (Jenei, 2025). Despite their legal basis, compulsory licensing efforts face intense political and economic pressures. In Colombia, attempts to issue a license for imatinib provoked direct intervention from the Swiss and U.S. governments, who warned about negative consequences for trade and investment (Leu, 2015). Similar dynamics have been focused between the U.S. and Brazil over HIV antiretrovirals, and in Thailand over the licensing of cancer medicines (Nagarajan, 2024). Countries pursuing TRIPS flexibilities have faced not only diplomatic and commercial retaliation, but even personal security threats. In one instance, a WHO employee overseeing pharmaceutical pricing policies was forced to live under police protection after facing security incidents across three continents (Nagarajan, 2024; Vidal, 2001). These cases underscore the high-stakes political environment surrounding the use of TRIPS flexibilities.

#### WHO prequalification and pooled procurement

WHO prequalification and pooled procurement are related mechanisms that can reduce prices by accelerating access to quality-assured generics. The WHO Prequalification Program ensures that medicines meet standards for quality, safety, and efficacy which in turn enables large buyers to engage in pooled procurement. This can lead to economies of scale that may drive down the prices of essential medicines (Figure 2). Global initiatives, such as the Global Fund, Gavi, and the Pan American Health Organization use the WHO listings, WHO guidelines, and prequalification to guide bulk purchasing decisions.

One study found that coordinated efforts between WHO guidelines, prequalification, and pooled procurement of major organizations such as the Global Fund, PEPFAR, and UNITAID, resulted in price decreases globally of over 80% for commonly used antiretroviral regimens between 2004 and 2008 (Waning et al., 2010). Furthermore, WHO prequalification may help overcome data exclusivities which prevent generic manufacturers from accessing the clinical data needed for regulatory approval. Another notable example is the Pan American Health Organization (PAHO) Strategic Fund, established in 2000, which facilitates pooled procurement of essential medicines and strategic health supplies across the Americas. The Fund provides countries with access to quality-assured medicines at lower prices, supports demand forecasting, and offers interest-free credit lines to bridge procurement cycles (Pan American Health Organization, 2025). Its long-standing role in improving access to antiretrovirals, oncology medicines, and other high-cost products (and connection to the WHO EML) illustrates the potential of pooled procurement to aid affordability barriers in diverse health system contexts.

However, limited coordination between WHO guidelines and the WHO EML (Piggott, Moja, Jenei, et al., 2024) reduces the ability of procurement agencies to prioritise medicines and shape markets. To better align listing with affordability, WHO could formally consider cost of production data during EML deliberations and enhance synergies between the EML, treatment guidelines, prequalification, and procurement agencies. This is particularly relevant for identifying high-priced medicines with potential for low-cost production (e.g. risdiplam) (Barber, 2023).

#### Price transparency and political advocacy

Political advocacy can be instrumental in price reductions and can help overcome resistance from pharmaceutical companies or governments. In 2013, WHO recognised ‘the potential political impact of identifying a medicine as essential for advocacy purposes’ under public health relevance criteria (World Health Organization, 2013). While this influence is not clearly defined, EML designation can symbolically elevate the importance of a medicine, or prompt political momentum, price negotiations (Figure 2).

This symbolic weight is reflected in the extensive commentary about EML inclusions and exclusions (Supplemental Table S1). For example, authors from LMICs viewed the inclusion of direct oral anticoagulants positively, despite high costs, as it signalled an impetus for national governments to prioritise their availability (Noubiap & Kamtchum-Tatuene, 2022). During the HIV crisis, advocacy around the exclusion and affordability of antiretrovirals from the WHO EML aligned with global financing architecture and contributed to reduced prices and improved access (Wachter, 1992). Recent initiatives have emerged to improve access to cancer medicines, such as Common Sense Oncology (Booth et al., 2023).

In 2019, 194 Member States adopted the WHO Transparency Resolution (WHA resolution 72.8) that urged public disclosure of net prices for health products, underscoring some political will to exert pressure on the pharmaceutical industry (World Health Assembly, 2019). However, accessing reliable production cost estimates (or actual manufacturer data) remains highly challenging, if not impossible. This lack of data limits the ability of governments and advocates to negotiate. International reference pricing has been one of the few tools available for cross-country comparisons. The MSH international reference prices were widely used for procurement and benchmarking. However, these data have not been updated since 2015, which limits their value today. Consolidated advocacy may also influence corporate behaviour. EML inclusion could serve as a public benchmark of pharmaceutical companies’ commitment to global access given corporate social governance becoming more relevant for shareholders. Examples of this include the Access to Medicines Index which rank companies based on various dimensions of access to their pharmaceutical products (Access to Medicines Foundation, 2025).

## Conclusion

The evolution of the WHO EML reflects the shifting priorities in global health. Once a tool for resource-constrained countries has evolved into a global standard that increasingly includes some high-priced medicines. However, this expansion has surfaced new challenges, such as balancing clinical benefit with prices and affordability. As a result, several effective medicines have been excluded from the Model Lists (Erfani et al., 2024). Key challenges include market and regulatory exclusivities that hinder generic competition, gaps in economic data, marginal clinical benefits for some therapies, and a persistent disconnect between market prices and production costs.

A range of mechanisms may help these barriers. Voluntary and compulsory licenses may enable earlier market entry of generic competitors. WHO Prequalification and pooled procurement may support collective bargaining to achieve medicine prices more aligned with production costs. In the absence of price decreases, countries (and individuals) may still exert political pressure through advocacy, including calls for greater price transparency and the use of international price comparisons in global health forums, such as the World Health Assembly or UN High Level Meetings, among others.

Future research should examine the impact of EML inclusion on medicine use and prices, including how mechanisms such as voluntary licenses and pooled procurement influence availability and affordability of medicines. Importantly, not all therapeutic areas behave similar in the market (e.g. ARVs versus cancer medicines), which may limit the interpretation from this analysis in certain areas. Additionally, understanding how countries interpret and apply the WHO EML can inform ongoing procedural reforms (World Health Organization, 2020). The relevance and credibility of the WHO EML in the current pharmaceutical ecosystem will depend on bridging the gap between inclusion on the Model Lists and availability at prices that are accessible to both patients and the health systems that serve them.

## Supplementary Material

Supplement.docx
