# Supplementary material for: Global mechanisms to improve affordability of high-priced medicines: the role of the WHO model lists of essential medicines
Source: J Pharm Policy Pract. 2026 Jan 9;19(1):2601935. doi: 10.1080/20523211.2025.2601935 (PMC12794694; doi:10.1080/20523211.2025.2601935)
Supplement: Supplement.docx [file JPPP_A_2601935_SM4429.docx]

**Supplementary File**

**Table S.1. Studies identified in the systematic literature review^a^**

| **Patents and essential medicines**   1. Attaran A. How do patents and economic policies affect access to essential medicines in developing countries? *Health Aff (Millwood)*. 2004;23(3):155-166. doi:[10.1377/hlthaff.23.3.155](https://doi.org/10.1377/hlthaff.23.3.155) 2. Baxi SM, Beall R, Yang J, Mackey TK. A multidisciplinary review of the policy, intellectual property rights, and international trade environment for access and affordability to essential cancer medications. *Globalization and Health*. 2019;15(1). doi:[10.1186/s12992-019-0497-3](https://doi.org/10.1186/s12992-019-0497-3) 3. Beall RF, Blanchet R, Attaran A. In which developing countries are patents on essential medicines being filed? *Global Health*. 2017;13(1):38. doi:[10.1186/s12992-017-0262-4](https://doi.org/10.1186/s12992-017-0262-4) 4. Loff B. Patents and access to essential drugs. *Transactions of The Royal Society of Tropical Medicine and Hygiene*. 2003;97(1):6-9. doi:[10.1016/S0035-9203(03)90002-2](https://doi.org/10.1016/S0035-9203(03)90002-2) 5. Luo J, Kesselheim AS. The Trans-Pacific Partnership Agreement and Implications for Access to Essential Medicines. *JAMA*. 2015;314(15):1563-1564. doi:[10.1001/jama.2015.10872](https://doi.org/10.1001/jama.2015.10872) 6. Nagarajan R. How many patients must die to pay the debt to drug innovation: Dr Melissa Barber. *The Times of India*. <https://timesofindia.indiatimes.com/home/sunday-times/all-that-matters/how-many-patients-must-die-to-pay-the-debt-to-drug-innovation-dr-melissa-barber/articleshow/115122367.cms>. November 10, 2024. Accessed November 12, 2024. 7. Shadlen KC, Sampat BN, Kapczynski A. Patents, trade and medicines: past, present and future. *https://doi-org.gate3.library.lse.ac.uk/101080/0969229020191624295*. 2019;27(1):75-97. doi:[10.1080/09692290.2019.1624295](https://doi.org/10.1080/09692290.2019.1624295) 8. ’T Hoen EFM. *The Global Politics of Pharmaceutical Monopoly Power: Drug Patents, Access, Innovation and the Application of the WTO Doha Declaration on TRIPS and Public Health*. AMB; 2009. 9. T’Hoen E, Berger J, Calmy A, Moon S. Driving a decade of change: HIV/AIDS, patents and access to medicines for all. *Journal of the International AIDS Society*. 2011;14(1):15-15. doi:[10.1186/1758-2652-14-15](https://doi.org/10.1186/1758-2652-14-15) 10. Ton I., Rahman T., Moon J., Jackevicius C. Essential and Evidence-Based Cardiovascular Disease Medicine Availability in Low-Cost Generic Drug Plans. *Circulation*. 2022;146. doi:[10.1161/circ.146.suppl_1.11314](https://doi.org/10.1161/circ.146.suppl_1.11314) 11. Van Puymbroeck RV. Basic survival needs and access to medicines--coming to grips with TRIPS: conversion + calculation. *J Law Med Ethics*. 2010;38(3):520-549. doi:[10.1111/j.1748-720X.2010.00510.x](https://doi.org/10.1111/j.1748-720X.2010.00510.x) 12. Velásquez G. Alternatives to the drug research and development model. *Salud Colect*. 2015;11(1):23-34. doi:[10.18294/sc.2015.413](https://doi.org/10.18294/sc.2015.413) 13. Zainol Z.A., Amin L., Jusoff K., Zahid A., Akpoviri F. Pharmaceutical patents and access to essential medicines in sub-Saharan Africa. *Afr J Biotechnol*. 2011;10(58):12376-12388. 14. Patently necessary: improving global access to essential medicines. *CMAJ*. 2003;169(12):1257. 15. IDF and Medicines Patent Pool welcome inclusion of SGLT2 inhibitors to WHO Essential Medicines List. *Diabetes Res Clin Pract*. 2021;180:109099. doi:[10.1016/j.diabres.2021.109099](https://doi.org/10.1016/j.diabres.2021.109099) |
| --- |
| **Global processes of the WHO EML**   1. Barbui C, Purgato M. Decisions on WHO’s essential medicines need more scrutiny. *BMJ*. 2014;349. doi:[10.1136/BMJ.G4798](https://doi.org/10.1136/BMJ.G4798) 2. Barr R.D. Essential medicines for children with cancer. *Pediatr Blood Cancer*. 2013;60(5):888. doi:[10.1002/pbc.24440](https://doi.org/10.1002/pbc.24440) 3. Beran D, Pedersen HB, Robertson J. Noncommunicable diseases, access to essential medicines and universal health coverage. *Glob Health Action*. 2019;12(1):1670014. doi:[10.1080/16549716.2019.1670014](https://doi.org/10.1080/16549716.2019.1670014) 4. Braine T. WHO to launch first essential medicines list for children. *Bull World Health Organ*. 2007;85(4):249-250. doi:[10.2471/BLT.07.010407](https://doi.org/10.2471/BLT.07.010407) 5. Brhlikova P, Deivanayagam TA, Babar ZUD, Osorio-de-Castro CGS, Caetano R, Pollock AM. Essential medicines concept and health technology assessment approaches to prioritising medicines: selection versus incorporation. *Journal of Pharmaceutical Policy and Practice*. 2023;16(1):88. doi:[10.1186/s40545-023-00595-4](https://doi.org/10.1186/s40545-023-00595-4) 6. Brhlikova P, Persaud N, Osorio-de-Castro CGS, Pollock AM. Essential medicines lists are for high income countries too. *BMJ*. 2023;382:e076783. doi:[10.1136/bmj-2023-076783](https://doi.org/10.1136/bmj-2023-076783) 7. Brundtland GH. Essential Medicines: 25 Years of Better Health. *JAMA*. 2002;288(24):3102. doi:[10.1001/jama.288.24.3102](https://doi.org/10.1001/jama.288.24.3102) 8. Cappello B., Moja L., Figueras A., Magrini N. The “Square Box”: Therapeutic Equivalence as a Foundation of the WHO Model List of Essential Medicines. *Front Pharmacol*. 2020;11:578000. doi:[10.3389/fphar.2020.578000](https://doi.org/10.3389/fphar.2020.578000) 9. Chirac P. Translating the essential drugs concept into the context of the year 2000. *Transactions of The Royal Society of Tropical Medicine and Hygiene*. 2003;97(1):10-12. doi:[10.1016/S0035-9203(03)90003-4](https://doi.org/10.1016/S0035-9203(03)90003-4) 10. Chirac P, Laing R. Updating the WHO essential drugs list. *Lancet*. 2001;357(9262):1134. doi:[10.1016/S0140-6736(00)04291-4](https://doi.org/10.1016/S0140-6736(00)04291-4) 11. Cohen D. Roche asks WHO to remove Avastin from essential medicines list. *BMJ*. 2017;356:j779. doi:[10.1136/bmj.j779](https://doi.org/10.1136/bmj.j779) 12. de Joncheere K. The role of the World Health Organization on pharmaceuticals in Europe. *Value Health*. 2001;4(3):217-224. doi:[10.1046/j.1524-4733.2001.43080.x](https://doi.org/10.1046/j.1524-4733.2001.43080.x) 13. Dugani S, Wasan KM, Kissoon N. World Health Organization and Essential Medicines. *J Pharm Sci*. 2018;107(5):1261-1262. doi:[10.1016/j.xphs.2017.12.019](https://doi.org/10.1016/j.xphs.2017.12.019) 14. Farrugia A. The World Health Organisation’s list of essential medicines and haemophilia treatment products. *Haemophilia*. 2023;29(6):1387-1389. doi:[10.1111/hae.14879](https://doi.org/10.1111/hae.14879) 15. Fernandez R., Sanchez D.S., Del Rio Torres H., Jane C.C. Essential medicines by the world health organisation and their convenience in elderly patients. *Euro J Hosp Pharm Sci Pra*. 2017;24:A244. doi:[10.1136/ejhpharm-2017-000640.545](https://doi.org/10.1136/ejhpharm-2017-000640.545) 16. Fojo T. The challenges of selecting cancer medicines for the WHO Essential Medicines List with the elephant in the room: A path forward. *Seminars in Oncology*. 2022;49(6):427-428. doi:[10.1053/j.seminoncol.2023.01.005](https://doi.org/10.1053/j.seminoncol.2023.01.005) 17. Gray AL, Wirtz VJ, ’T Hoen EFM, Reich MR, Hogerzeil HV. Essential medicines are still essential. *The Lancet*. 2015;386(10004):1601-1603. doi:[10.1016/S0140-6736(15)00514-0](https://doi.org/10.1016/S0140-6736(15)00514-0) 18. Greene JA. When did medicines become essential? *Bull World Health Organ*. 2010;88(7):483. doi:[10.2471/BLT.10.079970](https://doi.org/10.2471/BLT.10.079970) 19. Greene JA. Making medicines essential: The emergent centrality of pharmaceuticals in global health. *BioSocieties 2011 6:1*. 2011;6(1):10-33. doi:[10.1057/BIOSOC.2010.39](https://doi.org/10.1057/BIOSOC.2010.39) 20. Gulland A. WHO targets antimicrobial resistance in new essential medicines list. *BMJ*. 2017;357:j2809. doi:[10.1136/bmj.j2809](https://doi.org/10.1136/bmj.j2809) 21. Gupta S, Kumaravel J, Prakash A, Medhi B. Concept of essential medicines and recent updates. *Indian J Pharmacol*. 2023;55(1):1-5. doi:[10.4103/ijp.ijp_94_23](https://doi.org/10.4103/ijp.ijp_94_23) 22. Gupta S. Expanding the WHO list of essential medicines for children: A call for further action. *Pediatr Blood Cancer*. 2015;62(10):1685-1686. doi:[10.1002/pbc.25597](https://doi.org/10.1002/pbc.25597) 23. Hill SR, Gray A, Weber M. Setting standards for essential children’s medicines. *Bull World Health Organ*. 2007;85(9):650. doi:[10.2471/BLT.07.046151](https://doi.org/10.2471/BLT.07.046151) 24. Hogerzeil H.V., Hill S., Rago L. Boosting access to essential medicines for children. *Lancet*. 2006;368(9545):1419. doi:[10.1016/S0140-6736(06)69603-7](https://doi.org/10.1016/S0140-6736(06)69603-7) 25. Hogerzeil H.V., Liberman J., Wirtz V.J., et al. Promotion of access to essential medicines for non-communicable diseases: Practical implications of the un political declaration. *Lancet*. 2013;381(9867):680-689. doi:[10.1016/S0140-6736(12)62128-X](https://doi.org/10.1016/S0140-6736(12)62128-X) 26. Hogerzeil HV. The concept of essential medicines: lessons for rich countries. *BMJ : British Medical Journal*. 2004;329(7475):1169. doi:[10.1136/BMJ.329.7475.1169](https://doi.org/10.1136/BMJ.329.7475.1169) 27. Jenei K, Glaus CEG, Vokinger KN. WHO shapes priorities for medicines? An analysis of the applicants and decision makers within the historical evolution of the WHO Model Lists of Essential Medicines. *The Lancet*. 2024;404(10460):1365-1374. doi:[10.1016/S0140-6736(24)01549-6](https://doi.org/10.1016/S0140-6736(24)01549-6) 28. Kishore S, Herbstman B. Adding a Medicine to the WHO Model List of Essential Medicines. *Clinical Pharmacology & Therapeutics*. 2009;85(3):237-239. doi:[10.1038/clpt.2008.258](https://doi.org/10.1038/clpt.2008.258) 29. Laing R, Waning B, Gray A, Ford N, ’T Hoen E. 25 Years of the WHO essential medicines lists: Progress and challenges. *Lancet*. 2003;361(9370). doi:[10.1016/S0140-6736(03)13375-2](https://doi.org/10.1016/S0140-6736(03)13375-2) 30. Magrini N., Robertson J., De Joncheere K., Bero L. On WHO’s essential medicines process and transparency. *BMJ (Online)*. 2014;349:g5637. doi:[10.1136/bmj.g5637](https://doi.org/10.1136/bmj.g5637) 31. Magrini N, Robertson J, Forte G, Capello B, Moja L, Kieny MP. Tough decisions on essential medicines in 2015. *Bulletin of the World Health Organization*. 2015;93:283-284. 32. Magrini, Nicola. On WHO’s essential medicines process and transparency. Published online 2014. Accessed April 25, 2023. <https://www.bmj.com/content/349/bmj.g4798/rr/762816> 33. Maheshwari D.G., Jadav B.H. Global role of pre-qualification programme of essential medicines. *Int J Pharm Sci Rev Res*. 2015;31(1):212-216. 34. Manikandan S. Are we moving towards a new definition of essential medicines? *J Pharmacol Pharmacother*. 2015;6(3):123-125. doi:[10.4103/0976-500X.162008](https://doi.org/10.4103/0976-500X.162008) 35. Marks VA, Latham SR, Kishore SP. On Essentiality and the World Health Organization’s Model List of Essential Medicines. 2017;83(3-4):637. doi:[10.1016/j.aogh.2017.05.005](https://doi.org/10.1016/j.aogh.2017.05.005) 36. Motchane JL. Health for all or riches for some: WHO’s responsible? *Int J Health Serv*. 2003;33(2):395-400. doi:[10.2190/YL2E-H3VD-QJHQ-RKUL](https://doi.org/10.2190/YL2E-H3VD-QJHQ-RKUL) 37. Peacocke EF, Dale E, Mori AT, Koduah A, Gopinathan U. Measuring the value of the WHO Model list of essential medicines. *Bull World Health Organ*. 2024;102(10):684-684A. doi:[10.2471/BLT.24.292521](https://doi.org/10.2471/BLT.24.292521) 38. Perlino C, Daniel H, Cadwallader AB. Which Drugs Should Be on the Essential Medicines List? *AMA J Ethics*. 2024;26(4):E282-E288. doi:[10.1001/amajethics.2024.282](https://doi.org/10.1001/amajethics.2024.282) 39. Piggott T, Moja L, Akl EA, et al. Decision criteria for selecting essential medicines and their connection to guidelines: an interpretive descriptive qualitative interview study. *J Clin Epidemiol*. 2023;154:146-155. doi:[10.1016/j.jclinepi.2022.12.007](https://doi.org/10.1016/j.jclinepi.2022.12.007) 40. Piggott T, Moja L, Cuello Garcia CA, et al. User-experience testing of an evidence-to-decision framework for selecting essential medicines. *PLoS Global Public Health*. 2024;4(1). doi:[10.1371/journal.pgph.0002723](https://doi.org/10.1371/journal.pgph.0002723) 41. Piggott T, Moja L, Huttner B, et al. WHO Model list of essential medicines: visions for the future. *Bull World Health Organ*. 2024;102(10):722-729. doi:[10.2471/BLT.24.292359](https://doi.org/10.2471/BLT.24.292359) 42. Piggott T, Moja L, Jenei K, et al. GRADE Concept 7: Issues and Insights Linking Guideline Recommendations to Trustworthy Essential Medicine Lists. *J Clin Epidemiol*. 2024;166. doi:[10.1016/j.jclinepi.2023.111241](https://doi.org/10.1016/j.jclinepi.2023.111241) 43. Purgato M, Barbui C. What is the WHO essential medicines list? *Epidemiol Psychiatr Sci*. 2012;21(4):343-345. doi:[10.1017/S204579601200039X](https://doi.org/10.1017/S204579601200039X) 44. Quick JD. Essential medicines twenty-five years on: closing the access gap. *Health Policy and Planning*. 2003;18(1):1-3. 45. Quick JD, Hogerzeil HV, Velasquez G, Rago L. Twenty-five years of essential medicines. *Bull World Health Organ*. 2002;80(11):913-914. 46. Rehan H.S., Banerjee I., Suranagi U.S. Are we moving towards a new definition of essential medicines? *J Pharmacol Pharmather*. 2015;6(4):234-235. doi:[10.4103/0976-500X.171871](https://doi.org/10.4103/0976-500X.171871) 47. Reich MR. Essential drugs: economics and politics in international health. *Health Policy*. 1987;8(1):39-57. doi:[10.1016/0168-8510(87)90129-1](https://doi.org/10.1016/0168-8510(87)90129-1) 48. Reidenberg MM. Essential Medicines for the Whole World. *Clinical Pharmacology & Therapeutics*. 2007;82(5):500-503. doi:[10.1038/sj.clpt.6100388](https://doi.org/10.1038/sj.clpt.6100388) 49. Reidenberg MM. World Health Organization Program for the Selection and Use of Essential Medicines. *Clinical Pharmacology & Therapeutics*. 2007;81(4):603-606. doi:[10.1038/sj.clpt.6100106](https://doi.org/10.1038/sj.clpt.6100106) 50. Robertson J, Hill SR. The Essential Medicines List for a Global Patient Population. *Clinical Pharmacology & Therapeutics*. 2007;82(5):498-500. doi:[10.1038/SJ.CLPT.6100392](https://doi.org/10.1038/SJ.CLPT.6100392) 51. Robertson J, Barr R, Shulman LN, Forte GB, Magrini N. Essential medicines for cancer: WHO recommendations and national priorities. *Bull World Health Organ*. 2016;94(10):735-742. doi:[10.2471/BLT.15.163998](https://doi.org/10.2471/BLT.15.163998) 52. Roitberg F, Amaral T, Cherny NI, et al. Essential cancer medicines: adding feasibility to the magnitude of clinical benefit value chain. *ESMO Open*. 2023;8(5):101617. doi:[10.1016/j.esmoop.2023.101617](https://doi.org/10.1016/j.esmoop.2023.101617) 53. Simão M, Wirtz VJ, Al-Ansary LA, et al. A global accountability mechanism for access to essential medicines. *The Lancet*. 2018;392(10163):2418-2420. doi:[10.1016/S0140-6736(18)32986-6](https://doi.org/10.1016/S0140-6736(18)32986-6) 54. Smith MK, Tickell S. The essential drugs concept is needed now more than ever. *Transactions of The Royal Society of Tropical Medicine and Hygiene*. 2003;97(1):2-5. doi:[10.1016/S0035-9203(03)90001-0](https://doi.org/10.1016/S0035-9203(03)90001-0) 55. Urrutia J, Candidate P, Porteny Phd Candidate T, Daniels N. What does it mean to put new hepatitis C drugs on a list of essential medicines? Published online 2016. doi:[10.1136/bmj.i2035](https://doi.org/10.1136/bmj.i2035) 56. Welch C. The composition of WHO’s expert committee on essential medicines needs more scrutiny. *BMJ*. 2014;349:g5211. doi:[10.1136/bmj.g5211](https://doi.org/10.1136/bmj.g5211) 57. Wirtz VJ, Gray AL, Sharma S, Sun J, Hogerzeil HV. Refocusing the World Health Organization’s Model List of Essential Medicines on the needs of low and middle income countries. *BMJ*. 2024;385:e077776. doi:[10.1136/bmj-2023-077776](https://doi.org/10.1136/bmj-2023-077776) 58. Wirtz VJ, Hogerzeil HV, Gray AL, et al. Essential medicines for universal health coverage. *The Lancet*. 2017;389(10067):403-476. doi:[10.1016/S0140-6736(16)31599-9](https://doi.org/10.1016/S0140-6736(16)31599-9) |
| **Prices (or costs) of WHO essential medicines**   1. Alvarez-Uria G., Thomas D., Zachariah S., Byram R., Kannan S. Cost-analysis of the who essential medicines list in a resource-limited setting: Experience from a district hospital in India. *J Clin Diagn Res*. 2014;8(5). doi:[10.7860/JCDR/2014/7976.4352](https://doi.org/10.7860/JCDR/2014/7976.4352) 2. Barber M, Gotham D, Hill A. Potential price reductions for cancer medicines on the WHO Essential Medicines List. *European Journal of Cancer*. 2017;72:S119. doi:[10.1016/S0959-8049(17)30471-9](https://doi.org/10.1016/S0959-8049(17)30471-9) 3. Barber MJ, Gotham D, Bygrave H, Cepuch C. Estimated Sustainable Cost-Based Prices for Diabetes Medicines. *JAMA Network Open*. 2024;7(3):e243474. doi:[10.1001/jamanetworkopen.2024.3474](https://doi.org/10.1001/jamanetworkopen.2024.3474) 4. Furlow B. WHO Essential Medicines Committee spotlights unaffordable drugs. *The Lancet Oncology*. 2021;0(0). doi:[10.1016/S1470-2045(21)00575-1](https://doi.org/10.1016/S1470-2045(21)00575-1) 5. Gavaza P, Simoyi T, Makunike B, Maponga CC. The prices people pay for medicines in Zimbabwe. *Cent Afr J Med*. 2009;55(1):14-19. doi:[10.4314/cajm.v55i1-4.63635](https://doi.org/10.4314/cajm.v55i1-4.63635) 6. Gotham D., Barber M.J., Hill A.M. Estimation of cost-based prices for injectable medicines in the WHO Essential Medicines List. *BMJ Open*. 2019;9(9):e027780. doi:[10.1136/bmjopen-2018-027780](https://doi.org/10.1136/bmjopen-2018-027780) 7. Gray AL, Wirtz VJ, ’T Hoen EFM, Reich MR, Hogerzeil HV. Essential medicines are still essential. *The Lancet*. 2015;386(10004):1601-1603. doi:[10.1016/S0140-6736(15)00514-0](https://doi.org/10.1016/S0140-6736(15)00514-0) 8. Hwang TJ, Kesselheim AS, Vokinger KN. Reforming the World Health Organization’s Essential Medicines List: Essential but Unaffordable. *JAMA*. Published online October 24, 2022. doi:[10.1001/JAMA.2022.19459](https://doi.org/10.1001/JAMA.2022.19459) 9. Jenei K, Aziz Z, Booth C, et al. Cancer medicines on the WHO Model List of Essential Medicines: processes, challenges, and a way forward. *The Lancet Global Health*. 2022;0(0). doi:[10.1016/s2214-109x(22)00376-x](https://doi.org/10.1016/s2214-109x(22)00376-x) 10. Lang D.L., Zhao F.-L., Robertson J. Prevention of postpartum haemorrhage: Cost consequences analysis of misoprostol in low-resource settings. *BMC Pregnancy Childbirth*. 2015;15(1):305. doi:[10.1186/s12884-015-0749-z](https://doi.org/10.1186/s12884-015-0749-z) 11. Li DG, Najafzadeh M, Kesselheim AS, Mostaghimi A. Spending on World Health Organization essential medicines in Medicare Part D, 2011-15: Retrospective cost analysis. *The BMJ*. 2019;366. doi:[10.1136/BMJ.L4257](https://doi.org/10.1136/BMJ.L4257) 12. Molina-Salazar R.E., Gonzalez-Marin E., Carbajal-de Nova C. Competition and prices in the Mexican pharmaceutical market. *Salud Publica Mex*. 2008;50:S496-503. 13. Moucheraud C, Wirtz VJ, Reich MR. Evaluating the quality and use of economic data in decisions about essential medicines. *Bulletin of the World Health Organization*. 2015;93(10):693-699. doi:[10.2471/BLT.14.149914](https://doi.org/10.2471/BLT.14.149914) 14. Mujinja PGM, Mackintosh M, Justin-Temu M, Wuyts M. Local production of pharmaceuticals in Africa and access to essential medicines: “urban bias” in access to imported medicines in Tanzania and its policy implications. *Global Health*. 2014;10:12. doi:[10.1186/1744-8603-10-12](https://doi.org/10.1186/1744-8603-10-12) 15. Ngorsuraches S., Chaiyakan K. Equitable Prices of Single-Source Drugs in Thailand. *Appl Health Econ Health Policy*. 2015;13(4):389-397. doi:[10.1007/s40258-015-0165-6](https://doi.org/10.1007/s40258-015-0165-6) 16. Niens L.M. Impoverishing medicines: A cross-country comparison of the affordability of medicines. *Value Health*. 2009;12(7):A242. 17. Pablos-Mendez A, Gowda DK, Frieden TR. Controlling multidrug-resistant tuberculosis and access to expensive drugs: a rational framework. *Bull World Health Organ*. 2002;80(6):489-495. 18. Papola D, Ostuzzi G, Todesco B, et al. Updating the WHO Model Lists of Essential Medicines to promote global access to the most cost-effective and safe medicines for mental disorders. *The Lancet Psychiatry*. 2023;0(0). doi:[10.1016/S2215-0366(23)00176-1](https://doi.org/10.1016/S2215-0366(23)00176-1) 19. Perehudoff K, Toebes B, Hogerzeil H. A human rights-based approach to the reimbursement of expensive medicines. *Bull World Health Organ*. 2016;94(12):935-936. doi:[10.2471/BLT.15.166371](https://doi.org/10.2471/BLT.15.166371) 20. Shrivastava N., Frazier L., Gupta S., et al. Evidence-based cost estimation of essential medicines for pediatric cancer care in Peru. *J Clin Oncol*. 2023;41(16):e18533. doi:[10.1200/jco.2023.41.16_suppl.e18533](https://doi.org/10.1200/jco.2023.41.16_suppl.e18533) 21. Suresh Saravdekar SS, Shukla VK, Upadhya OP, Madhukar Rai MR, Kiran Giri KG. Implementation of principles of Pharmacoeconomics and Pharmacovigilance to achieve optimal financial and therapeutic benefits through WHO - essential medicine policy and adoption of NLEM-Based hospital formulary policy. *Journal of Family Medicine and Primary Care*. 2019;8(6):1987-1993. doi:[10.4103/jfmpc.jfmpc_287_19](https://doi.org/10.4103/jfmpc.jfmpc_287_19) 22. Syrett K. Essential but expensive? The World Health Organization, access to medicines and human rights. *Netherlands Quarterly of Human Rights*. 2019;37(2):139-156. doi:[10.1177/0924051919844373](https://doi.org/10.1177/0924051919844373) 23. Trapani D, Curigliano G. The global landscape of drug development of trastuzumab biosimilars. *Journal of Cancer Policy*. 2021;28:100273. doi:[10.1016/j.jcpo.2021.100273](https://doi.org/10.1016/j.jcpo.2021.100273) 24. Twagirumukiza M, Annemans L, Kips JG, Bienvenu E, Van Bortel LM. Prices of antihypertensive medicines in sub-Saharan Africa and alignment to WHO’s model list of essential medicines. *Trop Med Int Health*. 2010;15(3):350-361. doi:[10.1111/j.1365-3156.2009.02453.x](https://doi.org/10.1111/j.1365-3156.2009.02453.x) 25. Undela K. Assessment Of Prices Of Essential Medicines For Chronic Diseases Prevalent In The Asia Pacific Region. *Value in Health*. 2016;19(7):A460. doi:[10.1016/j.jval.2016.09.660](https://doi.org/10.1016/j.jval.2016.09.660) 26. Vasan A, Kim JY. Essential medicines pricing—reform needed. *The Lancet*. 2009;373(9659):191-193. doi:[10.1016/S0140-6736(08)61763-8](https://doi.org/10.1016/S0140-6736(08)61763-8) 27. Walgate R. Drug companies should cut prices for developing countries, says G8 report. *Bull World Health Organ*. 2003;81(1):72-73. |
| **Connecting essential (and/or high-priced) medicines with human rights**   1. Hogerzeil H.V. Human rights approach to health policy: The case of essential medicines. *Trop Med Int Health*. 2009;14:3. doi:[10.1111/j.1365-3156.2009.02352.x](https://doi.org/10.1111/j.1365-3156.2009.02352.x) 2. Hogerzeil HV, Samson M, Casanovas JV, Rahmani-Ocora L. Is access to essential medicines as part of the fulfilment of the right to health enforceable through the courts? *The Lancet*. 2006;368(9532):305-311. doi:[10.1016/S0140-6736(06)69076-4](https://doi.org/10.1016/S0140-6736(06)69076-4) 3. Hogerzeil HV. Essential medicines and human rights: what can they learn from each other? *Bull World Health Organ*. 2006;84(5):371-375. 4. Katrina Perehudoff S, Toebes B, Hogerzeil H. Essential Medicines in National Constitutions: Progress Since 2008. *Health Hum Rights*. 2016;18(1):141-156. 5. Perehudoff SK, Laing RO, Hogerzeil HV. Access to essential medicines in national constitutions. *Bull World Health Organ*. 2010;88(11):800. doi:[10.2471/BLT.10.078733](https://doi.org/10.2471/BLT.10.078733) 6. Sekalala S., Rawson B. The Role of Civil Society in Mobilizing Human Rights Struggles for Essential Medicines: A Critique from HIV/AIDS to COVID-19. *Health Hum Rights*. 2022;24(2):177-189. 7. Seuba X. A human rights approach to the WHO Model List of Essential Medicines. *Bull World Health Organ*. 2006;84(5):405-411. doi:[10.2471/BLT.04.019133](https://doi.org/10.2471/BLT.04.019133) 8. Smith M.J., Forman L., Parker M., Perehudoff K., Rawson B., Sekalala S. Should COVID-19 Vaccines Authorized for Emergency Use Be Considered “Essential” Medicines? *Health Hum Rights*. 2021;23(1):145-150. |
| **The inclusion and exclusion of medicines on the WHO EML**   1. Agarwal A, Huffman MD. Inclusion of Polypills for Prevention of Cardiovascular Disease in the 23rd World Health Organization Model List of Essential Medicines: A Significant Step Towards Reducing Global Cardiovascular Morbidity and Mortality. *Glob Heart*. 2024;19(1):24. doi:[10.5334/gh.1310](https://doi.org/10.5334/gh.1310) 2. Asadi-Pooya AA, Guekht A. Antiseizure medications in the World Health Organization list of “essential medicines.” *Epilepsia*. 2023;64(7):1951-1952. doi:[10.1111/epi.17649](https://doi.org/10.1111/epi.17649) 3. Bai L, Zhan Y, Zhou Y, et al. Evidence of clinical benefit of WHO essential anticancer medicines for children, 2011–2021. *eClinicalMedicine*. 2023;59. doi:[10.1016/j.eclinm.2023.101966](https://doi.org/10.1016/j.eclinm.2023.101966) 4. Balhara YPS. Time to include buprenorphine-naloxone combination in the WHO Model List of Essential Medicines. *J Opioid Manag*. 2013;9(4):237. 5. Balhara YPS. A curious case of the World Health Organization’s (WHO) approach on alcohol use disorders-inferences from the WHO list of essential drugs. *Addiction*. 2013;108(11):2030. doi:[10.1111/ADD.12312](https://doi.org/10.1111/ADD.12312) 6. Barbui C, Papola D, Todesco B, Gastaldon C, Ostuzzi G. Ground-breaking change to the mental health section of the WHO Model List of Essential Medicines: implications for low- and middle-income countries. *Epidemiol Psychiatr Sci*. 2024;33:e3. doi:[10.1017/S2045796024000040](https://doi.org/10.1017/S2045796024000040) 7. Beran D., Yudkin J.S. Looking beyond the issue of access to insulin: What is needed for proper diabetes care in resource poor settings. *Diabetes Res Clin Pract*. 2010;88(3):217-221. doi:[10.1016/j.diabres.2010.03.029](https://doi.org/10.1016/j.diabres.2010.03.029) 8. Beran D, Perrin C, Billo N, Yudkin JS. Improving global access to medicines for non-communicable diseases. *Lancet Glob Health*. 2014;2(10):e561-e562. doi:[10.1016/S2214-109X(14)70189-5](https://doi.org/10.1016/S2214-109X(14)70189-5) 9. Bhattarai MD. Combination anti-hypertensives in WHO essential medicine list. *Lancet*. 2005;366(9486):633-634. doi:[10.1016/S0140-6736(05)67133-4](https://doi.org/10.1016/S0140-6736(05)67133-4) 10. Breckenridge A. Debate that “This house believes the essential drug concept hinders the effective deployment of drugs in developing countries.” *Transactions of The Royal Society of Tropical Medicine and Hygiene*. 2003;97(1):1. doi:[10.1016/S0035-9203(03)90000-9](https://doi.org/10.1016/S0035-9203(03)90000-9) 11. Cesare MD, Jarvis JD, Scarlatescu O, et al. NOACs Added to WHO’s Essential Medicines List: Recommendations for Future Policy Actions. Published online 2020. doi:[10.5334/gh.774](https://doi.org/10.5334/gh.774) 12. Chiang CY, Trébucq A, Piubello A, Rieder HL, Van Deun A. Should gatifloxacin be included in the model list of essential medicines? *Eur Respir J*. 2018;51(2). doi:[10.1183/13993003.02329-2017](https://doi.org/10.1183/13993003.02329-2017) 13. Cleary J. Essential medicines in palliative care. *Palliat Med*. 2014;28(4):291-292. doi:[10.1177/0269216314527036](https://doi.org/10.1177/0269216314527036) 14. Cohen D. Lucentis excluded from WHO list of essential medicines. *BMJ*. 2015;350:h2573. doi:[10.1136/bmj.h2573](https://doi.org/10.1136/bmj.h2573) 15. Cortese S., Coghill D., Mattingly G.W., Rohde L.A., Wong I.C.K., Faraone S.V. WHO Essential Medicines List and methylphenidate for ADHD in children and adolescents - Authors’ reply. *Lancet Psychiatry*. 2024;11(2):93-95. doi:[10.1016/S2215-0366(23)00437-6](https://doi.org/10.1016/S2215-0366(23)00437-6) 16. Costa E, Moja L, Wirtz VJ, et al. Uptake of orphan drugs in the WHO essential medicines lists. *Bull World Health Organ*. 2024;102(1):22-31. doi:[10.2471/BLT.23.289731](https://doi.org/10.2471/BLT.23.289731) 17. De Lima L, Krakauer EL, Lorenz K, Praill D, Macdonald N, Doyle D. Ensuring palliative medicine availability: the development of the IAHPC list of essential medicines for palliative care. *J Pain Symptom Manage*. 2007;33(5):521-526. doi:[10.1016/j.jpainsymman.2007.02.006](https://doi.org/10.1016/j.jpainsymman.2007.02.006) 18. Denburg AE, Fundytus A, Khan MS, et al. Defining Essential Childhood Cancer Medicines to Inform Prioritization and Access: Results From an International, Cross-Sectional Survey. *JCO Global Oncology*. 2022;(8). doi:[10.1200/GO.22.00034](https://doi.org/10.1200/GO.22.00034) 19. Epstein J.S., Maryuningsih Y., Faber J.-C., Smid W.M., Burnouf T. Inclusion of cryoprecipitate, pathogen-reduced, in the WHO model lists of essential medicines for adults and children: a call for action. *Blood Transfus*. Published online 2024. doi:[10.2450/BloodTransfus.687](https://doi.org/10.2450/BloodTransfus.687) 20. Fernandez R., Sanchez D.S., Del Rio Torres H., Jane C.C. Essential medicines by the world health organisation and their convenience in elderly patients. *Euro J Hosp Pharm Sci Pra*. 2017;24:A244. doi:[10.1136/ejhpharm-2017-000640.545](https://doi.org/10.1136/ejhpharm-2017-000640.545) 21. Gibson L. WHO puts abortifacients on its essential drug list. *BMJ*. 2005;331(7508):68. 22. Gill G., Yudkin J.S., Tesfaye S., et al. Essential medicines and access to insulin. *Lancet Diabetes Endocrinol*. 2017;5(5):324-325. doi:[10.1016/S2213-8587(17)30090-6](https://doi.org/10.1016/S2213-8587(17)30090-6) 23. Gray NJ, Chanoine JP, Farmer MY, et al. NCDs and the WHO Essential Medicines Lists: children need universal health coverage too. *The Lancet Child & Adolescent Health*. 2019;3(11):756-757. doi:[10.1016/S2352-4642(19)30294-9](https://doi.org/10.1016/S2352-4642(19)30294-9) 24. Gulland A. WHO survey finds half of countries do not have clinical guidelines for treating hepatitis. *BMJ*. 2013;347:f4715. doi:[10.1136/bmj.f4715](https://doi.org/10.1136/bmj.f4715) 25. Hogerzeil HV. Rare Diseases and Essential Medicines. *Int J Pharm Med*. 2005;19(5):285-288. doi:[10.2165/00124363-200519050-00005](https://doi.org/10.2165/00124363-200519050-00005) 1. 26. Smith MK, Tickell S. The essential drugs concept is needed now more than ever. *Transactions of The Royal Society of Tropical Medicine and Hygiene*. 2003;97(1):2-5. doi:[10.1016/S0035-9203(03)90001-0](https://doi.org/10.1016/S0035-9203(03)90001-0) 27. Hogerzeil HV. Opposing the motion. *Transactions of The Royal Society of Tropical Medicine and Hygiene*. 2003;97(1):14-15. doi:[10.1016/S0035-9203(03)90005-8](https://doi.org/10.1016/S0035-9203(03)90005-8) 28. Hoppu K, Ranganathan SS. Essential medicines for children. *Archives of Disease in Childhood*. 2015;100(Suppl 1):S38-S42. doi:[10.1136/archdischild-2013-305705](https://doi.org/10.1136/archdischild-2013-305705) 29. Horton J. Debate that “This house believes the essential drug concept hinders the effective deployment of drugs in developing countries.” *Transactions of the Royal Society of Tropical Medicine and Hygiene*. 2003;97:12-16. 30. Huffman M.D., Yusuf S. Polypills: Essential medicines for cardiovascular disease secondary prevention? *J Am Coll Cardiol*. 2014;63(14):1368-1370. doi:[10.1016/j.jacc.2013.08.1665](https://doi.org/10.1016/j.jacc.2013.08.1665) 31. Hutchings J, Neroutsos K, Donnelly K. Making the List: The Role of Essential Medicines Lists In Reproductive Health. *IPSRH*. 2010;36(04):205-208. doi:[10.1363/3620510](https://doi.org/10.1363/3620510) 32. Hwang TJ, Kesselheim AS, Vokinger KN. Reforming the World Health Organization’s Essential Medicines List: Essential but Unaffordable. *JAMA*. Published online October 24, 2022. doi:[10.1001/JAMA.2022.19459](https://doi.org/10.1001/JAMA.2022.19459) 33. Joshi TP, Ren V. Essentiality and economy: a feasibility approach to evaluating suggested revisions to the World Health Organization Model List of Essential Medicines for skin disease. *Br J Dermatol*. 2021;185(5):1077. doi:[10.1111/bjd.20565](https://doi.org/10.1111/bjd.20565) 34. Kamerman PR, Wadley AL, Davis KD, et al. World Health Organization essential medicines lists: where are the drugs to treat neuropathic pain? *Pain*. 2015;156(5):793-797. doi:[10.1097/01.j.pain.0000460356.94374.a1](https://doi.org/10.1097/01.j.pain.0000460356.94374.a1) 35. Khan F.N., Hirsch I.B. Diabetes and the WHO Model List of Essential Medicines. *Lancet Diabetes Endocrinol*. 2022;10(1):16-17. doi:[10.1016/S2213-8587(21)00318-1](https://doi.org/10.1016/S2213-8587(21)00318-1) 36. Kishore SP, Bitton A, Cravioto A, Yach D. Enabling access to new WHO essential medicines: the case for nicotine replacement therapies. *Globalization and Health*. 2010;6(1):22. doi:[10.1186/1744-8603-6-22](https://doi.org/10.1186/1744-8603-6-22) 37. Kishore SP, Blank E, Heller DJ, et al. Modernizing the World Health Organization List of Essential Medicines for Preventing and Controlling Cardiovascular Diseases. *Journal of the American College of Cardiology*. 2018;71(5):564-574. doi:[10.1016/J.JACC.2017.11.056](https://doi.org/10.1016/J.JACC.2017.11.056) 38. Li Y, Yu J, Du L, et al. Exploration and practice of methods and processes of evidence-based rapid review on peer review of WHO EML application. *J Evid Based Med*. 2015;8(4):222-228. doi:[10.1111/jebm.12181](https://doi.org/10.1111/jebm.12181) 39. Logez SMD, Hutin YJF, Holloway K, Gray R, Hogerzeil HV. Could the WHO Model List of Essential Medicines Do More for the Safe and Appropriate Use of Injections? *The Journal of Clinical Pharmacology*. 2004;44(10):1106-1113. doi:[10.1177/0091270004268410](https://doi.org/10.1177/0091270004268410) 40. Machado IK, Lippert C, Ostacher MJ. Incorporating clinical research into recommendations for addressing global mental health needs: Updating the World Health Organization’s Essential Medicines List. *Compr Psychiatry*. 2021;109:152245. doi:[10.1016/j.comppsych.2021.152245](https://doi.org/10.1016/j.comppsych.2021.152245) 41. Mayor S. WHO includes 16 new cancer drugs on list of essential medicines. *The Lancet Oncology*. 2015;16(7):757. doi:[10.1016/S1470-2045(15)70240-8](https://doi.org/10.1016/S1470-2045(15)70240-8) 42. McKelvey D. Essential medicines lists in an era of escalating climate change. *BMJ*. 2023;383:2552. doi:[10.1136/bmj.p2552](https://doi.org/10.1136/bmj.p2552) 43. Millard C, Brhlikova P, Pollock A. Social networks and health policy: The case of misoprostol and the WHO model essential medicine list. *Social Science & Medicine*. 2015;132:190-196. doi:[10.1016/j.socscimed.2015.03.011](https://doi.org/10.1016/j.socscimed.2015.03.011) 44. Millard C, Brhlikova P, Pollock AM. Commentary: Evidence versus influence in the WHO procedure for approving essential medicines: misoprostol for maternal health. *BMJ*. 2014;349:g4823. doi:[10.1136/bmj.g4823](https://doi.org/10.1136/bmj.g4823) 45. Minghui R, Simao M, Mikkelsen B, Kestel D, Szilagyi Z. Gaps in access to essential medicines and health products for noncommunicable diseases and mental health conditions. Published online 2020:9-10. 46. Noubiap JJ, Kamtchum-Tatuene J. Addition of direct oral anticoagulants to the World Health Organization model list of essential medicines for the treatment of atrial fibrillation: An African perspective. *British Journal of Clinical Pharmacology*. 2022;88(7):3035-3038. doi:[10.1111/bcp.15226](https://doi.org/10.1111/bcp.15226) 47. Papola D., Ostuzzi G., Gastaldon C., Barbui C. The WHO Model List of Essential Medicines for Children needs its own identity: the case of psychotropic medicines. *Lancet Child Adolesc Health*. 2023;7(12):819-821. doi:[10.1016/S2352-4642(23)00220-1](https://doi.org/10.1016/S2352-4642(23)00220-1) 48. Papola D, Ostuzzi G, Todesco B, et al. Updating the WHO Model Lists of Essential Medicines to promote global access to the most cost-effective and safe medicines for mental disorders. *The Lancet Psychiatry*. 2023;0(0). doi:[10.1016/S2215-0366(23)00176-1](https://doi.org/10.1016/S2215-0366(23)00176-1) 49. Patel A, Vidula M, Kishore SP, Vedanthan R, Huffman MD. Building the Case for Clopidogrel as a World Health Organization Essential Medicine. *Circ Cardiovasc Qual Outcomes*. 2015;8(4):447-451. doi:[10.1161/CIRCOUTCOMES.115.001866](https://doi.org/10.1161/CIRCOUTCOMES.115.001866) 50. Pierce GF, O’Mahony B, Kaczmarek R, et al. Risk of harm to people with haemophilia from the 2023 WHO Essential Medicines List. *The Lancet Haematology*. 2024;0(0). doi:[10.1016/S2352-3026(24)00223-0](https://doi.org/10.1016/S2352-3026(24)00223-0) 51. Ranganathan S.S. Essential medicines list for children, what it means for the developing countries. *Basic Clin Pharmacol Toxicol*. 2010;107:37. doi:[10.1111/j.1742-7843.2010.00598-2.x](https://doi.org/10.1111/j.1742-7843.2010.00598-2.x) 52. Reidenberg M. Are drugs for rare diseases essential? *Bull World Health Organ*. 2006;84(9):686-686. doi:[10.2471/BLT.06.0034447](https://doi.org/10.2471/BLT.06.0034447) 53. Ribeiro J.P., Gluud C., Storm M.R.O., Storebo O.J. Should methylphenidate be included in the WHO model lists of essential medicines? *Eur Child Adolesc Psychiatry*. Published online 2024. doi:[10.1007/s00787-024-02565-w](https://doi.org/10.1007/s00787-024-02565-w) 54. Ribeiro JP, Lunde C, Gluud C, Simonsen E, Storebø OJ. Methylphenidate denied access to the WHO List of Essential Medicines for the second time. *BMJ Evidence-Based Medicine*. 2023;28(2):75-77. doi:[10.1136/bmjebm-2021-111862](https://doi.org/10.1136/bmjebm-2021-111862) 55. Rimmer K, Shah H, Thakur K. Expanding medicines for neurologic disorders on the WHO Model List. *Neurology*. 2017;88(10):e87-e91. doi:[10.1212/WNL.0000000000003691](https://doi.org/10.1212/WNL.0000000000003691) 56. Robertson J, Magrini N, Barr R, Forte G, Ondari C. Medicines for cancers in children: The WHO model for selection of essential medicines. *Pediatr Blood Cancer*. 2015;62(10):1689-1693. doi:[10.1002/pbc.25564](https://doi.org/10.1002/pbc.25564) 57. Rundle CW, Fortugno AP, Maghfour J, et al. Evaluating the World Health Organization Model List of Essential Medicines for skin disease. *British Journal of Dermatology*. 2021;185(2):451-453. doi:[10.1111/BJD.20081](https://doi.org/10.1111/BJD.20081) 58. Scott C., Smith N., James R., Whitehead B., Green R., Foster H. Revising the who essential medicines list for paediatric rheumatology. *Pediatr Rheumatol*. 2020;18. doi:[10.1186/s12969-020-00470-5](https://doi.org/10.1186/s12969-020-00470-5) 59. Seetharaman R. Inclusion of drugs on the World Health Organization’s Essential Medicines List: A game-changer for obesity? *Diabetes Obes Metab*. 2023;25(8):2423-2424. doi:[10.1111/dom.15114](https://doi.org/10.1111/dom.15114) 60. Shulman L., Wagner C., Torode J., et al. Establishment of a New Approach to Defining Essential Medicines for Cancer: Implications of the 2014-2015 UICC Review of the WHO Model List. *JCO Glob Oncol*. 2016;2(3):47s-48s. doi:[10.1200/JGO.2016.004374](https://doi.org/10.1200/JGO.2016.004374) 61. Shulman LN, Wagner CM, Barr R, et al. Proposing Essential Medicines to Treat Cancer: Methodologies, Processes, and Outcomes. *J Clin Oncol*. 2016;34(1):69-75. doi:[10.1200/JCO.2015.61.8736](https://doi.org/10.1200/JCO.2015.61.8736) 62. Singu BS, Verbeeck RK. Should codeine still be considered a who essential medicine? *Journal of Pharmacy and Pharmaceutical Sciences*. 2021;24:329-335. doi:[10.18433/JPPS31639](https://doi.org/10.18433/JPPS31639) 63. Soyannwo OA. Improved neuropathic pain treatment in developing countries--a critical review of WHO essential list. *Pain*. 2015;156(5):763-764. doi:[10.1097/j.pain.0000000000000140](https://doi.org/10.1097/j.pain.0000000000000140) 64. Stolk P, Willemen MJC, Leufkens HGM. “Rare essentials”: Drugs for rare diseases as essential medicines. *Bulletin of the World Health Organization*. 2006;84(9):745-751. doi:[10.2471/BLT.06.031518](https://doi.org/10.2471/BLT.06.031518) 65. Storebo O.J., Gluud C. Methylphenidate for ADHD rejected from the WHO Essential Medicines List due to uncertainties in benefit-harm profile. *BMJ Evid Based Med*. 2021;26(4):172-175. doi:[10.1136/bmjebm-2019-111328](https://doi.org/10.1136/bmjebm-2019-111328) 66. The Lancet Haematology. Essential medicines: a balancing act. *The Lancet Haematology*. 2019;6(12):e597. doi:[10.1016/S2352-3026(19)30238-8](https://doi.org/10.1016/S2352-3026(19)30238-8) 67. Unguru Y., Bernhardt M.B., Berg S.L., et al. Essential medicines for childhood cancer in Europe. *Lancet Oncol*. 2023;24(2):e67. doi:[10.1016/S1470-2045(23)00009-8](https://doi.org/10.1016/S1470-2045(23)00009-8) 68. Wertheimer AI, Santella TM. Innovation and the WHO’s essential medicines list: Giving credit where credit is due. *Research in Social and Administrative Pharmacy*. 2007;3(1):137-144. doi:[10.1016/j.sapharm.2006.05.006](https://doi.org/10.1016/j.sapharm.2006.05.006) 69. Yamout BI, Viswanathan S, Laurson-Doube J, Sokhi D. Disease-modifying therapies enter the World Health Organization Essential Medicines List: A victory now requiring a roadmap of implementation. *Mult Scler*. 2024;30(1):3-6. doi:[10.1177/13524585231205970](https://doi.org/10.1177/13524585231205970) 70. Zaidel E.J., Leng X., Adeoye A.M., et al. Inclusion in the world health organization model list of essential medicines of non-vitamin k anticoagulants for treatment of non-valvular atrial fibrillation: A step towards reducing the burden of cardiovascular morbidity and mortality. *Glo Heart*. 2020;15(1):52. doi:[10.5334/GH.608](https://doi.org/10.5334/GH.608) 71. Zhou Y, Naci H, Chen D, et al. Overall survival benefits of cancer drugs in the WHO Model List of Essential Medicines, 2015–2021. *BMJ Global Health*. 2023;8(9):e012899. doi:[10.1136/bmjgh-2023-012899](https://doi.org/10.1136/bmjgh-2023-012899) |
| **On access to essential medicines generally**^a^   1. Ahmad K. Access denied to essential medicines in developing world. *Lancet Infect Dis*. 2002;2(12):711. doi:[10.1016/s1473-3099(02)00466-8](https://doi.org/10.1016/s1473-3099(02)00466-8) 2. Alpern JD, Song J, Stauffer WM. Essential Medicines in the United States — Why Access Is Diminishing. *New England Journal of Medicine*. 2016;374(20):1904-1907. doi:[10.1056/NEJMp1601559](https://doi.org/10.1056/NEJMp1601559) 3. Armstrong K. It’s not “all too hard”! Together we CAN improve access to essential paediatric endocrine medicines for all. A roadmap to 2030. *Horm Res Paediatr*. 2023;96:7. doi:[10.1159/000529083](https://doi.org/10.1159/000529083) 4. Bazargani Y.T., de Boer A., Schellens J.H.M., Leufkens H.G.M., Mantel-Teeuwisse A.K. Essential medicines for breast cancer in low and middle income countries. *BMC Cancer*. 2015;15(1):591. doi:[10.1186/s12885-015-1583-4](https://doi.org/10.1186/s12885-015-1583-4) 5. Bazargani YT, Ewen M, De Boer A, Leufkens HGM, Mantel-Teeuwisse AK. Essential Medicines Are More Available than Other Medicines around the Globe. *PLOS ONE*. 2014;9(2):e87576. doi:[10.1371/JOURNAL.PONE.0087576](https://doi.org/10.1371/JOURNAL.PONE.0087576) 6. Cameron A, Ewen M, Ross-Degnan D, Ball D, Laing R. Medicine prices, availability, and affordability in 36 developing and middle-income countries: a secondary analysis. *Lancet*. 2009;373(9659):240-249. doi:[10.1016/S0140-6736(08)61762-6](https://doi.org/10.1016/S0140-6736(08)61762-6) 7. Chirac P, Laing R. Updating the WHO essential drugs list. *Lancet*. 2001;357(9262):1134. doi:[10.1016/S0140-6736(00)04291-4](https://doi.org/10.1016/S0140-6736(00)04291-4) 8. Cohen P., Friedrich P., Lam C., et al. Global access to essential medicines for childhood cancer: A cross-sectional survey. *J Glob Oncol*. 2018;2018(4):1-11. doi:[10.1200/JGO.18.00150](https://doi.org/10.1200/JGO.18.00150) 9. Dionisio D., Gass R., McDermott P., et al. What strategies to boost production of affordable fixed-dose anti-retroviral drug combinations for children in the developing world? *Curr HIV Res*. 2007;5(2):155-187. doi:[10.2174/157016207780077075](https://doi.org/10.2174/157016207780077075) 10. Eden T., Burns E., Freccero P., et al. Are essential medicines available, reliable and affordable in low-middle income countries? *J Cancer Policy*. 2019;19:100180. doi:[10.1016/j.jcpo.2018.12.001](https://doi.org/10.1016/j.jcpo.2018.12.001) 11. Eom G, Grootendorst P, Duffin J. The case for an essential medicines list for Canada. *CMAJ*. 2016;188(17):E499-E503. doi:[10.1503/cmaj.160134](https://doi.org/10.1503/cmaj.160134) 12. Gray A., Holloway K. Is WHO’s essential medicines list contributing to rational medicines use in developing countries? *Basic Clin Pharmacol Toxicol*. 2010;107:7-8. doi:[10.1111/j.1742-7843.2010.00598-1.x](https://doi.org/10.1111/j.1742-7843.2010.00598-1.x) 13. Gupta R, Kim JY, Espinal MA, et al. Public health. Responding to market failures in tuberculosis control. *Science*. 2001;293(5532):1049-1051. doi:[10.1126/science.1061861](https://doi.org/10.1126/science.1061861) 14. Jenei K, Wirtz VJ. Measuring access to essential medicines in the sustainable development goals. *Bull World Health Organ*. 2024;102(8):555-555A. doi:[10.2471/BLT.24.291399](https://doi.org/10.2471/BLT.24.291399) 15. Kraus R, Yeung RSM, Persaud N. Biologic medicine inclusion in 138 national essential medicines lists. *Pediatric Rheumatology*. Published online 2021. doi:[10.1186/s12969-021-00608-z](https://doi.org/10.1186/s12969-021-00608-z) 16. Levy M, Reidenberg MM. What has been the impact of the concept of essential drugs? *Clinical Pharmacology & Therapeutics*. 2003;73(4):275-278. doi:[10.1016/S0009-9236(03)00003-1](https://doi.org/10.1016/S0009-9236(03)00003-1) 17. Mahmić-Kaknjo M, Jeličić-Kadić A, Utrobičić A, Chan K, Bero L, Marušić A. Essential medicines availability is still suboptimal in many countries: a scoping review. *Journal of Clinical Epidemiology*. 2018;98:41-52. doi:[10.1016/j.jclinepi.2018.02.006](https://doi.org/10.1016/j.jclinepi.2018.02.006) 18. Meyer-Aandrieux I., Roberts T., Gaspani S., Milani B., Cohn J. Increasing access to diagnosis and treatments for hepatitis C (HCV) in resource limited settings (RLS), how should we move forwards? *J Hepatol*. 2013;58:S404. doi:[10.1016/S0168-8278(13)60982-4](https://doi.org/10.1016/S0168-8278(13)60982-4) 19. Millar TP, Wong S, Odierna DH, Bero LA. Applying the essential medicines concept to US preferred drug lists. *Am J Public Health*. 2011;101(8):1444-1448. doi:[10.2105/AJPH.2010.300054](https://doi.org/10.2105/AJPH.2010.300054) 20. Neumann I, Schünemann HJ, Bero L, Cooke G, Magrini N, Moja L. Global access to affordable direct oral anticoagulants. *Bull World Health Organ*. 2021;99(9):653-660. doi:[10.2471/BLT.20.278473](https://doi.org/10.2471/BLT.20.278473) 21. Petricca K, Carson L, Kambugu J, Denburg A. Strengthening access to cancer medicines for children in East Africa: policy options to enhance medicine procurement, forecasting, and regulations. *Glob Health Res Policy*. 2024;9(1):24. doi:[10.1186/s41256-024-00365-y](https://doi.org/10.1186/s41256-024-00365-y) 22. Quick JD. Ensuring access to essential medicines in the developing countries: a framework for action. *Clin Pharmacol Ther*. 2003;73(4):279-283. doi:[10.1016/s0009-9236(03)00002-x](https://doi.org/10.1016/s0009-9236(03)00002-x) 23. Rojo P. Access to essential drugs in developing countries. *Gac Sanit*. 2001;15(6):540-545. 24. Rowlands A, Deeb A, Ladjouze A, et al. Access to fludrocortisone and to hydrocortisone in children with congenital adrenal hyperplasia in the WHO Eastern Mediterranean Region: it takes a village. *BMJ Global Health*. 2021;6(10). doi:[10.1136/bmjgh-2021-007195](https://doi.org/10.1136/bmjgh-2021-007195) 25. Seneviwickrama M, Gunasekera S, Liyanage G, Heiyanthuduwa W, Jayakody S. Availability of cytotoxic medicines in the WHO essential medicine list used in treating childhood malignancies in low-income and lower-middle-income countries: a systematic review protocol. *BMJ Open*. 2023;13(6):e071988. doi:[10.1136/bmjopen-2023-071988](https://doi.org/10.1136/bmjopen-2023-071988) 26. Shimazawa R., Ikeda M. Approval status and evidence for WHO essential medicines for children in the United States, United Kingdom, and Japan: A cross-sectional study. *J pharm policy pract*. 2017;10(1):4. doi:[10.1186/s40545-016-0094-2](https://doi.org/10.1186/s40545-016-0094-2) 27. Wilmshurst JM, Blockman M, Argent A, et al. Leaving the party -- withdrawal of South African essential medicines. *S Afr Med J*. 2006;96(5):419. 28. Wouters OJ, Kuha J. Low- And Middle-Income Countries Experienced Delays Accessing New Essential Medicines, 1982-2024. *Health Aff (Millwood)*. 2024;43(10):1410-1419. doi:[10.1377/hlthaff.2024.00089](https://doi.org/10.1377/hlthaff.2024.00089) 29. Zucker H, Rägo L. Access to essential medicines for children: the world health organization’s global response. *Clin Pharmacol Ther*. 2007;82(5):503-505. doi:[10.1038/sj.clpt.6100395](https://doi.org/10.1038/sj.clpt.6100395) |

**^a^**There is a large subset of research on access, affordability, and availability of essential medicines at country-level, often using the Health Action International/WHO methodology. These studies have not been itemised in this list given the focus on global processes. However, a systematic review of these studies has been included (Cameron, 2009). Several studies overlap across categories as they address multiple points within their analyses.
